# Virtual fashion experiences in virtual reality fashion show spaces

**DOI:** 10.3389/fpsyg.2023.1276856

**Published:** 2023-11-17

**Authors:** Se Jin Kim

**Affiliations:** Department of Clothing and Textiles, Changwon National University, Changwon, Republic of Korea

**Keywords:** virtual fashion experience, fashion show, virtual fashion space, cognitive presence, sensible immersion, emotional immersion, aesthetic interaction

## Abstract

**Introduction:**

Virtual reality (VR) provides a new fashion space and fashion experience. This study focuses on immersive VR and fashion shows to empirically explore the VR fashion space and fashion experience. Insights specific to fashion have not been presented in as much depth in the literature; thus, the current findings are particularly valuable and insightful.

**Methods:**

This study employed three immersive VR (IVR) fashion show stimuli and in-depth interviews according to a semi-structured questionnaire. Collected data were analyzed based on the concept of VR space and VR experience derived through literature research.

**Results:**

The VR fashion space was divided into three types and VR experiences of cognitive presence, sensible immersion, emotional immersion, and aesthetic interaction were derived accordingly. First, the physical representation of a fashion show induced a cognitive and emotional sense of presence, in which users felt as though they had moved to the same time and place as those at the fashion show. Second, participants experienced cognitive confusion owing to the differences with *a priori* experiences in the fashion show space (i.e., reality and imagination coexist). Third, participants transcended the limitations of physical reality while in the fashion show space of pataphysics (which was realized with human imagination), and they moved beyond the stage of confusion that is experienced while facing realistic objects to connect to creative inspiration.

**Discussion:**

The difference in the properties of VR space may be associated with distinct VR fashion experiences. The findings suggest that (1) *a priori* elements such as sociocultural contexts and personal experiences differ in the experiential dimension of virtual space, (2) the VR fashion show space induces a psychological experience between brand and consumer, and (3) creative inspiration and exploratory play can be greatly induced in a user if the immersive fashion space is further from the original source.

## 1 Introduction

Humans accumulate experience when interacting with space, which is a medium through which the world is perceived (Tuan, 2011). Space is signified through dynamic interrelationships between itself and the various elements that constitute it (Lefebvre, 1991). As space is not just a static, physical background, attention needs to be paid to its active aspects.

Virtual reality (VR) spaces provide humans with virtual spaces, with social functions that are similar to space in reality (Barreda-Ángeles and Hartmann, 2022). For example, VR can facilitate social interactions between people who are geographically distant, enabling collaboration. Humans accumulate novel experiences in this new space. A VR space can provide an environment that is either similar to reality or one that is completely new. The space allows users to feel an immediate sense of immersion, even more so than the sense of presence in the real world and physical reality, and this virtual human experience is real (Pelet et al., 2017). Therefore, the experiential impact that the new digital environment has on humans needs to be determined.

VR affects fashion shows, which are a site for the presentation of fashion and serve as a means of communication between fashion brands and audiences. Space has not been given the same consideration as clothes vis-à-vis fashion shows. Yet, fashion shows cannot be held without space. It is an important component in developing and conveying the concept and image of a fashion collection (Mendes, 2019). Various places have been used effectively to convey the fashion show’s image and concept (Strömberg, 2019). VR technology allows users to access fashion shows at any time and from any place. Advances in communication infrastructure such as the Internet of Things and 5G technology support this, and a Metaverse world that can be accessed from anywhere in the world is opening up. VR spaces based on the properties of digital media share the characteristics of imagined and physical images (Wideström, 2019). They transcend regional, cultural, and temporal boundaries and have unique spatial characteristics that cannot be experienced in a physical space. Thus, VR fashion show spaces can affect the fashion experiences of users.

Fashion studies have focused on topics such as the use of virtual environment technology in the development of fashion stores and designs and the effect they have on user experience. Park et al. (2018) showed that VR fashion stores have a positive impact on users’ shopping behavior vis-à-vis enjoyment and purchase intention. Sina and Wu (2023) revealed that the appropriate use of design elements in VR fashion stores leads to high results regarding consumers’ emotions, perceptions, and satisfaction levels. Lighting color and color temperature in a 360-degree VR space are also related to consumers’ shopping motivation. Jung et al. (2021) noted that expanding the experience of a luxury fashion show (which had been allowed to a small, limited audience previously) to the public would popularize luxury items.

These findings are significant as they suggest that human perception and experience of the fashion industry can be affected by the relevant VR technology. However, a fashion show differs from a fashion store with regards to purpose, content, and experience. Fashion shows provide fashion images, communication, and entertainment. VR fashion show spaces allow various interactions between brands, designers, and users to create new meaning for fashion shows and novel fashion experiences. Therefore, investigating such content is meaningful as it will expand on previous studies to present a new perspective.

This study focused on immersive VR environments and students majoring in fashion. Immersive VR (IVR) uses wearable devices such as head-mounted displays (HMDs; Shahrbanian et al., 2012). Ricci et al. (2023) noted that IVR was associated with higher hedonic and utilitarian values, and better user experience when compared to general VR. IVR provides higher levels of sense of presence, immersion, and emotion than does general VR (Kerrebroeck et al., 2017). The experiential dimension could differ based on user characteristics; users with higher levels of openness to experience (Costa and McCrae, 1997; which is a prominent characteristic of artists) experience a deeper aesthetic experience through the sense of presence in VR (Starkey et al., 2021). Therefore, fashion students are required to have emotional and sensory experiences that will enhance their IVR experiences compared to general students.

This study investigated users’ fashion experiences in IVR fashion show spaces, in which VR and an HMD are used. VR fashion show spaces refer to the virtual realm in which fashion shows are conducted using IVR technology. This study explored the following research questions: First, how are VR spaces and experiences defined? Second, what kind of fashion experiences do users have in VR fashion shows? A framework of analysis was prepared through a literature review for research question 1. For research question 2, in-depth interviews were conducted with undergraduate fashion students with higher levels of immersion to analyze users’ virtual fashion experiences through VR fashion shows.

## 2 Literature review

### 2.1 The VR space as an experience-creating medium

#### 2.1.1 Perspectives and characteristics of space

Space is the realm or world in which certain substances and objects can exist or events can occur and is simultaneously universal and abstract. It is a medium that defines human thoughts and forms individuals’ unique experiences (Buttimer and Seamon, 2015). Space is formed in the relationship between an object and the human who perceives and recognizes it (Ashihara, 1981).

Experiences are formed during a human’s interactions with a given space. This sociological approach to space explains the interactions between people, space, and human behavior rather than focusing on the meaning of physical backgrounds. This approach is useful in establishing a conceptual framework to unpack human experiences in VR space.

There are many discussions on space from a sociological perspective. The first is the space of physical interactivity. Space is not an abstract entity that is distinct from the objects existing within it; it can only be understood through concrete events or objects (Choi, 2016). Löw and Weidenhaus (2017) defined space as a relational arrangement of social goods and living beings existing in places. Space does not exist independently. It is formed by exchanges that take place among the sociocultural background, and the actors and products within it. Therefore, space is created by social institutions and actors and cannot exist independently from these elements. Simmel (2005) paid attention to the kinds of spatial experiences that people undergo in a city, and the cognitive functions that take place, and found that the meaning of space is created, and interactions are amplified through human engagement. Space is nothing in itself but becomes full through interactions and gains meaning vis-à-vis individuals and social groups (Schroer, 2010).

The second is the space of fluidity and dynamism. Modern society is flexible and fluid because of its fast pace and advancements in information technology. Bauman (2005) distinguished this from the solidity of the modern era and described the spatial characteristics of modern society as being liquid. He considered a city an intensely liquid space that is not fixed in one space and is consistently moving owing to the loose solidarity between the individualized elements constituting the liquid. Advancements in information technology cause changes in social patterns and create new spatial logics called the Space of Flow (space–time created by the fluidity of changes among social institutions, cultures, and members; Castells, 2010). Therefore, space is not fixed and eternal, but rather expands, integrates, and fractures in its relationship with changing social institutions, cultural contexts, and members.

The third is the space of symbolism. Foucault presented the concept of heterotopia; for example, everyday spaces such as public baths, prisons, and colonies embody either deviant ideals within the institutions of modern society or incompatible utopian concepts (Foucault, 1967). This interpretation disproves the idea that space has a symbolic meaning. Lefebvre (1991) considered space a social product and stated that it can control or influence human consciousness and behavior. The framework of social space was based on trialectics, which includes representations of space as perceived through human activities, cognitive space such as lifestyles, and space of representations that is experienced with a combination of symbols and images (Lefebvre, 1991). It is a view of space as a social product and its representation differs from the second perspective in terms of the formative identity of space. Soja (1989) stated that space is formed as material spatial practices that reproduce the actual form and concrete patterns of lifestyles, which are transformed into an imaginary conceived space that becomes conceptual and is expressed symbolically, changing into a lived space that is structured by the experiences of individuals or groups. These discussions suggest that space is created by humans, the agents of action, and can be symbolically realized through interactions with social institutions. Thus, space is a result of human interaction and a social construct.

Accordingly, this study approaches the spatial characteristics of VR space by considering the properties of digital space in the next chapter based on the three perspectives regarding the concept of space.

#### 2.1.2 Approach to fashion shows and VR spaces

By reviewing previous studies on digital media spaces (Choi and Park, 2019; Suh, 2020) to determine their characteristics, this study identified the following: ambiguity and extensibility of space–time boundaries; complexity and flexibility of information; virtuality of reality; interactions that enable two-way communication between information providers and users; and ease of access to information. These characteristics are classified as follows by comprehensively considering the properties of space examined earlier (Table 1).

**Table 1:** The characteristics of VR space.

Social space	Digital media space	VR space
Physical interaction	- Interaction - Ease of access	Space of physical representation
Fluidity and dynamism	- Complexity and flexibility of information - Ambiguity and extensibility of space-time boundaries	Mixed space of reality and idea
Symbolism	Virtuality	Space of pataphysics

First, the space of physical representation is the space in which the real world’s physical appearance is projected and reconstructed. Although VR space is artificial, it is reproduced by reflecting reality (Lombard et al., 2006; Deng et al., 2019). New media interacts with old media (Bolter and Grusin, 2000). Second, the mixed space of reality and imagination is the space in which reality and virtuality coexist, which is characterized by the flexibility and dynamism of information, and in which space and time are not clearly separated. Modern space has a complex and fluid character and breaks away from the existing fixed concept of space and time owing to the rapid exchange of information and advances in the media (Bauman, 2005). Digital media spaces are characterized by a property of variability that differs from physical space and old media (Manovich, 2001). Therefore, the VR space has a mixed character that encompasses all these properties, in which reality and virtuality coexist owing to the variability and fluidity of digital images. For example, while components of the traditional fashion show reproduce the space–time of reality in the VR space, they construct a new fashion show space by creating an ideal object or by transforming the existing appearance. Third, the space of pataphysics is based on symbolic creation. The term pataphysics was proposed by Alfred Jarry and refers to an area of study that extends beyond metaphysics, and to the virtual nature of things based on imagination (Bok, 2002). Space in pataphysics cannot exist in reality but is realistically created by human imagination. Modern society is a world of simulacra in which reality has been replaced by symbols (Baudrillard, 1994). This VR space is the space of symbolic ideals in which symbolic objects are realized without material substance. The spatial characteristics of pataphysics are significant in the image-oriented fashion industry as they can express images that designers want to convey without limitations and communicate with users.

### 2.2 VR experience

Humans accumulate new experiences by interacting with various elements surrounding digital media. For example, digital images have a color range different with nature, which means it gives humans supernormal stimulation. VR technology also provides opportunities to travel to outer space and other places. As an example of VR fashion show, the 21FW Balenciaga collection—set in universe and in the form of a 3D virtual video game—was presented and audiences could easily access it anytime, anywhere.

The VR resulting from digital media is a 3D environment that is reproduced to enable physical experiences as in the real world. In the VR space, users can navigate the space as they do in real life, interact with objects, and experience one or more of their five senses being stimulated in real time (Burdea and Coiffet, 2003). While more recent studies (Freeman et al., 2019; Rathinam et al., 2019) have focused on the possibility of VR technology to help human psychological treatment and physical rehabilitation, there is a lack of research that identifies emotional and aesthetic experiences, especially in fashion studies, regarding the circumstances of the advanced IVR environment.

Thus, this study explored the virtual fashion experience through the concept of space and virtual experience. For this, a framework was built through a literature review about the concept of space as its focus, previous studies on the relationship between brands and users, and relevant studies on VR experiences, summarizing the results (Table 2).

**Table 2:** The dimension of VR experience in VR space.

Brand experience	VR experience		VR experience in VR space
Perception	Presence	Authenticity	Cognitive presence
Sense			
			Sensible immersion
	Immersion		
Emotion		Interaction	Emotional immersion
	Emotion Simulation		Aesthetic interaction
Behavior			

#### 2.2.1 Cognitive presence

The sense of presence refers to the psychological experience of feeling as though you are in a specific space in the virtual world or perceiving created objects, people, and events as being real (Lombard et al., 2006). The sense of presence can be divided according to the degree of interaction between the user and virtual space and media. Among the subdivisions, the cognitive sense of presence refers to the experience of perceptual illusion, in which the user mistakenly feels as though it is a real physical environment or object owing to the physical stimulations provided by digital media. This is a cognitive experience in which the user feels they are physically present in a specific space and perceives various information through sensory stimulations (Biocca and Levy, 2000); the cognitive sense of presence causes a user to experience place illusion, in which they feel as though they are in a specific place by experiencing the virtual space even though they are not actually present (Slater, 2009).

As a sense of presence is based on sensory information, it increases when the media provides more detailed and elaborate information as if in real life (Lombard and Ditton, 1997). It is classified as the characteristics of media systems that generate a sense of presence concerning vividness and interactivity (Steuer, 1992). If high and intense levels of sensory information are acquired in the VR space, it causes high levels of vividness (Li et al., 2002). Interactivity refers to the degree to which a user can affect the content and form of a mediated environment in real time, and it is determined by the speed at which user inputs are reflected, as well as their range, and how natural the mapping is (Steuer, 1992). For example, naturalness in a VR environment is related to high fidelity and it enhances the sense of presence (McMahan et al., 2012). Therefore, interactivity can be increased by high levels of fidelity in the VR space, in which elaborate, realistic, and sensory expressions are possible.

Although not much research on the effect of interaction in the VR space exists, there have been some studies on VR related to sports (Sigrist et al., 2015; Vogt et al., 2015). According to them, the sense of presence is related to the electroencephalogram and was highest in the interactive VR condition, showing that interactive VR produces physical performance in a highly competitive spirit. However, the impact of the high fidelity of VR fashion presentation and user experience has not been proven yet.

#### 2.2.2 Sensible immersion

Immersion refers to the state in which the user’s perceptual and psychological awareness are almost completely cut off from the outside world while participating in VR, and the pleasurable experience of being transported to an elaborately simulated place, alongside the sensory, emotional experience of being surrounded by a completely different reality that takes over their attention and sense organs (Murray, 1997). It is the flow that is experienced owing to external stimuli, and it is primarily used to describe the experience of using VR devices (Cheng et al., 2017). This study examined the characteristics of immersion by distinguishing between sensory and emotional immersion. Sensory immersion refers to the range of sensory channels involved in virtual simulation (Kim and Biocca, 2018). Using purely sensory receptors such as one’s sight and hearing to acquire information also increases the immersion effect when compared to using one type of sensory channel. It can further focus on the sensory properties of the garment as an object. Thus, this study aimed to determine whether users obtain tactile effects in non-contact situations of VR.

#### 2.2.3 Emotional immersion

Emotion is a personal response to internal or external stimuli that is caused by a somewhat specific cause or event; it provides information on a specific object or situation (Payne and Cooper, 2001). Sensory organs sense external stimuli and the sensed information is transmitted to the brain, which undergoes mental processes called emotions and reactions. The implicit memories of a person, psychological experiences, feelings that are expressed as emotions through external physical stimuli, and the resulting high-level emotional responses are sensibilities (Han and Choi, 2020). A user can experience human senses and emotions through the VR space, without directly induced stimuli and events, which is the plausibility illusion (Slater, 2009). Although sensibilities and emotions can be discussed separately, this study uses the term emotion to collectively define psychological responses that are distinct from perception. Users of the VR space experience emotional immersion through objects and emotions such as joy and sadness (Han and Choi, 2020).

Emotional immersion is closely related to sensory experience. The sense of space and distance are related to creating emotional experiences in themselves (Diemer et al., 2015; Peperkorn et al., 2015). Facial expressions are effective in inducing emotions (Han, 2018), and the hyper-realistic expression of digital humans in virtual space induces interest and pleasure; through this satisfaction, the level of immersion increases owing to intrinsic rewards and the sense of self-achievement, even if there are no special goals or extrinsic rewards (Kim and Seo, 2017).

Thus, visual expressions bring about emotional immersion. In fashion presentations, emotional communication such as fashion images is important. This study revealed how users gain virtual fashion experiences through the emotional immersion provided by the IVR environment.

#### 2.2.4 Aesthetic interaction

The aesthetic experience refers to an attitude, perception, experience, or interest related to art appreciation (Wanzer et al., 2020). “Aesthetic” refers to a sensory experience that is related to the visual form, texture, harmony, order, and beauty of an artifact (Venkatesh and Meamber, 2008), and is an ideal experience characterized by an aesthetic quality that is distinct from everyday experiences (Marković, 2012). This experience arises from the dynamic interactions between the perceptions of objects and processing of perceived information and is formed with the addition of judgment and evaluation (Venkatesh et al., 2010). It is influenced by culture, reference groups, and personal taste (McCracken, 2005).

Aesthetic experience is not only limited to exhibitions and works of art; it is also found in various areas of activity such as in sports, games, and exploration (Csikszentmihalyi and Robinson, 1990). It refers to the element of pleasure that is based on the understanding of the object. Norman (2002) stated that the aesthetic experience in the goods and services system includes the immediate sense of the object and the joy and pleasure that are experienced in the process of comparing the characteristics to the information saved in one’s memory.

An aesthetic interaction refers to the dynamic exchange of information, such as product characteristics and sensory and cognitive experiences that include user behavior between the person and artifact contexts (Locher et al., 2010). Instead of being seen as a unilateral experience, an aesthetic interaction should be considered an active and agentic concept in which information is shared between humans and objects. Kang (2023) analyzed the aesthetic experience of the VR exhibition and showed that the IVR exhibition was highly evaluated in the emotional and intellectual factors than the VR exhibition. Ma et al. (2023) showed that education using VR technology can foster children’s aesthetic creativity. These provide important grounds suggesting that IVR can affect aesthetic experience and creativity. Nevertheless, the aesthetic experience of fashion presentation in an IVR environment has not been considered so far.

The experience of aesthetic interactions is appropriate to define this complex experience as a separate characteristic for examining the user’s experience of VR fashion show spaces. This is because each experiential element in the VR space can appear in a complex manner unlike in real spaces, and because the nature of fashion, unlike other fields, has aesthetics that reflect the internal experience of external objects. This study examined the experience of aesthetic interactions in the VR space separately concerning experiences such as the sensory and emotional pleasure regarding objects, and the psychological state of satisfaction with fashion collections and brands as consumer goods.

## 3 Materials and methods

### 3.1 Participants

Interviews were conducted in a controlled laboratory on campus. Denzin and Lincoln (2003) argued that content was more important than the number of cases and suggested no more than 10 cases. Consequently, this study selected 21 participants, with 7 per stimulus (Table 3). Participants were recruited through purposive and snowball sampling. Recruitment notices were distributed through social media and in-depth interviews were conducted with respondents. According to Lee and Park (2018), those who prefer communicating with new communication technologies are in their 20s and 30s. Thus, this study selected male and female participants in South Korea who were in their 20s, majoring in fashion/clothing, and had experience watching fashion shows or videos, but had no experience with VR. Those who satisfied the necessary conditions and expressed their intention to participate were selected (Table 3). This study received ethics approval from an institutional review board (no. 7001066-202301-HR-003).

**Table 3:** Participants.

No.	Age (years)	Sex	Stimuli
1	26	M	S1
2	21	M	S1
3	24	M	S1
4	26	M	S1
5	21	F	S1
6	27	F	S1
7	23	F	S1
8	22	F	S2
9	23	F	S2
10	22	F	S2
11	25	F	S2
12	25	F	S2
13	25	M	S2
14	24	F	S2
15	23	F	S3
16	24	F	S3
17	22	F	S3
18	27	M	S3
19	22	F	S3
20	23	F	S3
21	23	F	S3

All were undergraduates majoring in clothing and textiles except for no. 6 (postgraduate in fashion design). No one had any VR experience.

### 3.2 Stimuli and devices

Of the case selection methods suggested by Jason and John (2008), a typical method was used to select the most representative cases, which were 360-degree VR fashion shows that had the three types of characteristics vis-à-vis virtual space. The following method was used to select the stimuli: In January 2023, keywords combining “360,” “VR,” and “fashion show” were entered on YouTube VR to search for fashion shows of different brands that had the highest number of likes and that clearly showed each of the three characteristics of virtual space. Accordingly, three videos were selected. Fashion show spaces comprise models, runway clothes, music, lighting, stage, venue, and the audience (Kim and Ahn, 2016; Kim, 2018). Accordingly, stimuli were selected by considering whether the elements of the traditional fashion show were reproduced, transformed, and maintained; these stimuli were created according to the characteristics of a VR space when the above material elements of the fashion show were converted to digital images.

Stimulus 1, Dior’s 2017 Spring/Summer Haute Couture Show, was produced by recording the live fashion show so that participants could watch the show as a 360-degree video from an audience perspective. The concept of this show was a labyrinth, and the space was constructed using grass and trees inside a tent that was set up in the Musée Rodin Garden (Mower, 2017). It was a fashion show space that reproduced the appearance of physical reality. Stimulus 2, Prada’s 2021 Spring/Summer Womenswear Show, was held without an audience and comprised a stage that was blocked off on all sides by digital screens and curtains. As with Stimulus 1, this space had a variable and flexible appearance as the viewer’s perspective was not fixed in place and the background moved at different stages. Digital screens were set up on the walls of the space to create another fashion show scene. It was a mixed space comprising a blend of reality and virtuality. Stimulus 3, TTSWTRS’s Technological Singularity Show, was produced in 2020. This fashion show displayed the transformation of objects, animals, and human figures into virtual objects and backgrounds that were made using digital images. A space of manufactured objects is one of pataphysics, which is expressed beyond the limitations of reality through interactions between the human imagination and VR technologies.

Although there are several types of IVR display devices, Oculus Quest 2 was used from among the wireless HMD devices that allow a wearer to move freely. This device was selected because it had a reduced weight and more comfortable, ergonomic design when compared to previous devices that could be attached to the wearer’s head. Released in 2020 by Meta, the Oculus Quest 2 comprises a VR headset and controller, and the screen can be moved while wearing the VR headset, by using the controller and neck movements.

### 3.3 Detailed interview questions

For feelings such as the sense of presence that users experience in VR, many consider that subjective statements are the standard method of measurement (Ijsselsteijn et al., 2000). Therefore, this study conducted in-depth interviews and used the participants’ statements for data analysis. Semi-structured questionnaires were used to facilitate detailed conversations in an open environment without any constraints (Karanika and Hogg, 2010). The questionnaire comprised items that could determine the characteristics of the VR experience. Items were referenced from previous studies on experience (Pinker, 1997; Shedroff, 2001; Mascarenhas et al., 2006), VR experience and space (Ji et al., 2022), and the sense of presence and immersion in VR environments (Steuer, 1992; Narciso et al., 2019; Kim and Choo, 2023) (Table 4).

**Table 4:** Detailed interview questions.

Item	Question	
Basic question	□ Age, occupation, education level, residence, use of social and digital media, and experience watching fashion shows □ Reason for watching fashion shows and what is expected of them	
Dimension of experience	Perception	□ What did you learn from watching the fashion show? □ What had you known about the brand and design previously? □ How does it compare to watching a fashion show on social media?
	Sense	□ Did the models and objects in the video feel three-dimensional? Why do you think so? □ How was the visual texture of the runway outfits, models, audience, etc.? □ How was the music in the fashion show space? □ How were the form and visual texture of the stage, background, and props? Please describe them in detail.
	Emotion	□ How did you feel after watching the fashion show? □ Did any images come to mind? Why is that? □ What was the most memorable thing after watching the video and how did it make you feel? Why is that?
	Behavior	□ How was the process of putting on the head-mounted display? □ Did you move your body while watching the video? If so, how did you move and why?
	Aesthetic	□ Has the image of the brand or design changed from what you originally experienced before? If so, how? □ Did you find anything particularly interesting while watching the video? Why is that? □ Please describe the physical characteristics or the impressions of what you saw in the fashion show space such as the models, stage, background, and runway outfits. Was anything particularly impressive? Why is that?
Sense of presence		□ Did you feel like you were in the fashion show space with other people and objects? Why is that? □ Did you feel like you were in the fashion show space?
Immersion		□ Did you ever forget that you were in this place (laboratory)? □ Did you feel like you could touch the models, stage, or the audience around you in the video? If you felt a similar feeling, please describe it. Why is that?

### 3.4 In-depth interview and methods of data collection and analysis

In a qualitative study, it is important to create a favorable atmosphere by building rapport with participants so that they can express themselves freely (Boyce and Neale, 2006). Two moderators who were students majoring in clothing and textiles helped build rapport with participants to create a relaxed atmosphere and encourage them to speak so that they would voluntarily share and interact with each other. In-depth interviews were conducted in the following manner: (1) Before watching the stimuli, participants were asked simple questions about themselves; (2) Participants who were going to experience VR were given an explanation on using the HMD, and a simple simulation was conducted to help them understand the device and learn how to operate it; and (3) After watching the stimuli (for approximately 3 to 4 min), participants were interviewed for 30 min using the prepared questionnaire. Their responses were recorded and transcribed using Naver CLOVA NOTE, an automatic recording program. The responses were moved to Microsoft Word for refinement and preparation of the data. Data were analyzed using qualitative methods. Participants’ responses were recorded in writing and compared with the key concepts and characteristics that were derived through the literature review. Data analysis was conducted in line with Giorgi (2004), where: (1) the content of the descriptions was understood in full, (2) the content of participants’ descriptions were organized into meaningful units, (3) the organized words were transformed into academic expressions and the themes and central meanings were determined, and (4) the central meanings were identified and categorized into groups.

## 4 Results

### 4.1 Virtual fashion experience representing space-time

#### 4.1.1 Cognitive sense of the place

In Stimulus 1, which comprised a 360-degree video of a real-life show, participants described experiencing place illusion, in which they felt as though they were with the audience in a specific place in which the fashion show was being held.

“The background was green and felt like a field. It felt like the spectators were sitting on a path in the field, and it was so realistic that I felt I was there” (Participant 7).

“If I turned my head just a little, I could see people right next to me talking to and looking at each other, so it felt like I was with the audience…It felt like I was in Paris so it somehow felt like I was dreaming!” (Participant 4).

These responses demonstrate that with the reproduction of the stage, background, and audience in the VR fashion show space, participants were fully aware that it was a fashion show. Rather than focusing on the details of elements such as the runway outfits and models, they were fascinated by the atmosphere in the space, and experienced the cognitive sense of presence, wherein they felt as though they were in the venue, which was actually in another region.

#### 4.1.2 Assimilation into the fashion show

In the space of Stimulus 1, participants were reminded of brands or images they had seen on other visual media, and fashion images were formed in addition to the atmosphere of the fashion show that was provided by the VR space.

“The location of the fashion show felt like a spring garden, and the floral atmosphere felt a lot like old-fashioned dresses” (Participant 6).

“There were a lot of dresses, and I thought of the image of a spring picnic” (Participant 4).

“The brand generally had a luxurious and elegant image” (Participant 7).

Participant 7 linked the fashion image to that of the brand. The VR fashion show space is a container in which fashion images are created through the interactions of each component and the user’s overall *a priori* experience, perception, and emotional response. As the space of a reproduced fashion show captures the appearance of a specific reality, participants had the emotional experience of forming fashion images based on previous experiences and then relating them to the brand identity. Participants said that they felt a sense of belonging to the brand as it felt as though they had attended a fashion show that was only open to a few people.

“I looked around a lot because it felt like I was experiencing a fascinating fashion show in the forest. I was surprised to see that famous actors were there, too!” (Participant 3).

“It felt novel and exclusive. I felt like I was watching the fashion show after getting a VIP seat invitation from the director” (Participant 1).

Experiencing a fashion show in which a specific time and place was reproduced created a sense of closeness among all the elements that shared the same space and time. This is a state of brand attachment that was formed in the fashion show space, wherein one created an emotional bond in the way they would with someone close to them (Thomson et al., 2005). It represented an emotional experience.

#### 4.1.3 Contextual understanding of runway outfits

During the fashion show, participants could see three-dimensional runway outfits and perceive their form, color, and texture. However, instead of remembering the outfits in detail, they appeared to try to understand the outfits in the context of the fashion show space.

“I saw a female model, and I was impressed because the stage and outfit that the model was wearing matched so well!” (Participant 7).

“It was nice to be able to see environmentally friendly outfits that fit the atmosphere of the fashion show” (Participant 3).

“It was different from the style of the brands I know, but it was good in that it showed various sides and reflected the changes by the trend” (Participant 1).

These responses contrasted the purpose of watching a fashion show, as participants had articulated in the beginning of the interview. Participants who were majoring in clothing and textiles stated that they occasionally watched fashion shows to gain information on outfits. However, in the reproduced VR space, participants were more immersed in the space and atmosphere than in the clothes and demonstrated the aesthetic experience of trying to understand the outfits in the context of the space.

The VR fashion show space, which is a physical representation of the original in real life, allows users to experience a representation of reality. Through this representational space, the user shows that they are forming an emotional bond by observing the fashion show in the context of brand identity and the sociocultural context that they perceive in reality and being immersed rather than focusing on fashion information.

### 4.2 Virtual fashion experience of mixed reality and virtuality

#### 4.2.1 Cognitive confusion

Participants experienced cognitive confusion, in which all the objects constituting this mixed space were considered artificially created virtual objects.

“I was mistaken about whether seeing real three-dimensional models was virtual reality or reality. I looked into the model’s eyes, and even though they were a fake person, I got goosebumps because they looked real” (Participant 10).

In Stimulus 2, some objects that comprised the digital screens and stage were created virtually. However, the models, runway outfits, and stage were real. Participants could not tell the difference. These responses suggest that although an enclosed virtual space that is blocked from the outside world can increase the psychological experience of immersion for users, it can cause them to experience cognitive confusion (where reality is considered virtual) if users are not given sufficient clues that relate to the physical reality.

#### 4.2.2 Sensory and emotional immersion of closeness

As with perception, the process of judging a situation begins with the senses (Lokesh et al., 2022). In the VR fashion show space, the movements of models and perspectives caused sensory experiences such as the three-dimensional effect and sense of distance. The elements of a fashion show, such as a model’s walk, differ from other mediums of fashion presentation as dynamism is emphasized. In the case of VR fashion shows, the dynamism provides a three-dimensional effect and a sense of space.

“I felt a strong sense of three-dimensionality. When the model came back, I felt like I could touch them if I reached out, and I felt like I was in the fashion show space with them” (Participant 9).

“The models made eye contact with me as they walked by, and it seemed like they were really looking at me and that the model was aware of me” (Participant 10).

Visual elements that are presented can have an effect on inducing emotions (Diemer et al., 2015; Peperkorn et al., 2015). An approach that reflects the sense of space and distance concerning visual perception acts to induce emotions; facial expressions also induce emotions (Han, 2018). As they felt that the models were walking by them, participants said that they experienced a sense of three-dimensional space and presence. Some said that they felt scared or a sense of familiarity because of the models’ eyes or expressions.

“The models’ eyes and expressions seemed a bit three-dimensional, and they felt real as they came closer. I was overwhelmed when I saw the models’ eyes and the fashion together” (Participant 10).

“When they approached me, I could feel it so vividly, like they were going to reach me right away, and it felt like having a friend right in front of me!” (Participant 8).

Regarding the eyes of digital humans, the addition of mutual interactivity, emotional expressions, visual processing technology, and biological characteristics can increase levels of immersion in visual immersion, self-purposefulness, concentration, and external insensitivity (Yun et al., 2020). Participants’ responses demonstrate that when the model’s eyes faced them and they came closer, this induced emotional experiences such as a sense of fear or familiarity. Although Stimuli 1 and 3 had dynamism, which is a component of fashion shows, when there is not enough information on the space (that is, when it is difficult to understand the space in the sociocultural context of reality), this suggests that there could be higher levels of emotional experience concerning the fashion objects.

#### 4.2.3 Authenticity of a fashion image

The new information and emotional experiences that are acquired in the VR fashion show space have an impact on the perceived image of the fashion brand. Participants recalled the fashion image of the brand they had known and explained that experiencing a VR fashion show that mixed reality and imagination either renewed their brand image or strengthened the existing one.

“I think the feeling of models walking up and making eye contact with me made me focus on the show more, and made me feel good because it felt like they were holding the show just for me” (Participant 8).

“It did not have that feeling that was characteristic to the brand. It had a very bright feeling…The show rather felt friendlier and a little different” (Participant 10).

Participants did not stop with these emotional experiences. They went on to connect them with their evaluation of the fashion brand. This is connected to fidelity, which is a measure of how well virtual environments represent the real world (Meyer et al., 2012), and is also strongly related to realism (Bowman and McMahan, 2007). In the VR fashion show space, in which reality and virtuality are mixed, participants said that low levels of fidelity increased virtuality and created doubts regarding brand originality.

“The overall quality of the fashion show I watched on YouTube was very high…but this somehow did not feel like Prada” (Participant 14).

It appears that participants felt more confused about the brand identity when they were not sufficiently provided with information that is generally well-known.

“As there wasn’t a logo or anything to symbolize the brand, I did not think anyone would know that they were that brand’s clothing” (Participant 10).

“I do not think I ever felt like it was a real stage…so I really did not think it was that brand” (Participant 11).

The VR fashion show space (in which there is a mix of reality and imagination) creates a new space–time that has fluidity and dynamism, and is a mix of the real and virtual. This fashion show space makes it difficult to understand objects based on the user’s past experiences, which is the sociocultural context of reality. Therefore, participants experienced immersion that was dependent on sensory elements, and as a result, the technology of graphics such as fidelity affected the authenticity of objects. This way, a fluid space can become an obstacle for users who are trying to immerse themselves in a brand (that is, trying to maintain the relationship between a brand and consumer; Gundlach et al., 1995), which is because the solidarity with the history, tradition, and identity that the brand built in reality weakens in this space. However, it is meaningful as it creates aesthetic experiences in which fashion images (that are strengthened or decreased) can be perceived or experienced through individual subjective insight and new spaces.

### 4.3 Virtual fashion experience of pataphysics

#### 4.3.1 Connection to creative inspiration

Although participants experienced a sense of presence as though they were in the same fashion show space as others, they considered the objects they observed unrealistic because they had neither seen nor touched them and could not touch them outside of the show. As a result of this “unreality,” participants said that they became curious about how the elements of an ideal fashion show space are created, and said that they felt inspired.

“As the video moved with me when I turned my head and moved my gaze, it really felt like I was in a fashion show…First, I felt it was a little unrealistic because the video’s composition and flow were unrealistic, and I had never experienced it and could not touch it” (Participant 21).

“I wonder how the graphics were implemented so well…It makes me wonder how long it must have taken to make this great video with the designs…I thought that it could be expressed in a more fun way if it was done this way” (Participant 16).

Before watching, participants majoring in the field of fashion stated that they ordinarily acquired new ideas and information through fashion shows. These responses demonstrate that unfamiliar images can provide new visual stimulation and cognitive experiences to a greater degree than when compared to other stimuli; this shows that they could provide material for creative inspiration concerning academic and work-related activities.

#### 4.3.2 Aesthetic exploratory play

Exploratory behavior obtains information on stimuli. Exploration is divided into specific exploration, in which a new object is manipulated and tested using one’s senses, and diverse exploration, which is a long-term and continuous exploration that takes place internally after an object’s characteristics have been identified (Hutt, 1971). Unlike exploratory behaviors, which are driven by external stimuli, playing is a multifaceted behavior that is driven by intrinsic motivation. It takes place after the information has been acquired and feelings of pleasure are induced in the process, and not in the results. With exploratory play, a combination of exploration and play takes place. It refers to the behavioral pattern that appears after a new object is observed based on an *a priori* experience related to the said object. The unfamiliar emotions that participants experienced in Stimulus 3 resulted in the behavior of collecting information by moving the field of view to various angles. Through this experience, participants explained that they became more curious about the brand’s concept and level of completeness of the content. This is similar to the exploratory play that young children engage in while exploring a new world.

“It was the first time I had seen a model or clothes three-dimensionally, so it felt like I was on a new amusement park ride” (Participant 21).

Participants’ physical activity in the VR fashion show space was limited to their eye movements. However, they were seen to acquire audiovisual information with virtual objects regardless of material achievements and physical behaviors. Even if participants could not use their will to control the situation directly, they used the information they acquired by guessing the connectivity between the fashion show’s components to see the fashion show as a work of art and understand its concept, which was an aesthetic exploratory experience.

The fashion show space of pataphysics, which was realized with human imagination, is an imaginary space that comprises a combination of images that are somewhat far from the experiential symbolism of lifestyles and social groups. This space (where the relationship between the social signifiers and the signified are disconnected) provides aesthetic experiences in which users engage in new exploratory play and dynamic interactions take place between fashion images and the users’ aesthetic exploration.

## 5 Conclusion

### 5.1 Summary and discussion

This study approached the VR space with three characteristics based on the perspective of social space and proposed that differences in spatial character led to differences in the fashion experiences of users.

First, the fashion show space, which is a physical representation, induced a cognitive and emotional sense of presence, in which users felt as though they had moved to the same time and place as those of the fashion show. Through this fashion show space, users experienced aesthetic interactions in the cultural context, in which runway outfits were interpreted by connecting them to the atmosphere of the representational space. Jung and Ko (2023) explored the experiences of luxury fashion brands and determined that the components of the virtual fashion space are related to virtual experiences such as immersion, presence, and interaction. *A priori* elements such as sociocultural contexts and personal experiences differ in the experiential dimension of virtual space. Stimulus 1 was the reproduction of a real luxury brand fashion show, and it was determined that the user became immersed by connecting the fashion images that were accumulated in reality to the virtual space. This immersion played a large role in forming the emotional bond that is referred to as a sense of belonging.

Second, in the mixed fashion show space (in which reality and imagination coexist), participants experienced cognitive confusion owing to the differences with *a priori* experiences. Participants’ sensory experience was connected to the formation of the brand’s image and emotional experiences. The three-dimensionality and a sense of space that occurs in enclosed spaces provided by VR technology induce a psychological experience in which the user feels as though they have a special and intimate relationship with the brand. Given that the stimuli are fashion shows of luxury brands, it is unusual that an emotional experience of intimacy is induced concerning a private relationship, which is unlike the sense of belonging in a representational space. Jung et al. (2021) argued that VR technology reflects a utopia in which traditional luxury fashion shows (that had been characterized by privilege and status in the past) can be experienced equally. This could lead to different consequences based on the properties of the space.

Third, participants transcend the limitations of physical reality while in the fashion show space of pataphysics (which was realized with human imagination) and that they move beyond the stage of confusion that is experienced while facing realistic objects to connect to creative inspiration. Unlike other spaces, the fashion show space of pataphysics is far from social symbolic meanings and is a space that results from the creator’s imagination. In virtual space, which is difficult to understand in the sociocultural context of reality, the user engaged in active exploratory behavior to acquire new information and underwent aesthetic experiences concerning aesthetic exploration. This confirmed the results of Kim and Choo (2023), who found that experiences of shopping in VR increased levels of perceptual curiosity and creativity in consumers. Research findings suggest that creative inspiration and exploratory play can be induced in a user to a greater degree if the immersive fashion space is further from the original source and if the relationship between the signifiers and the signified is further disconnected.

### 5.2 Implications and limitations

Studies in the field of fashion that have dealt with fashion experiences through IVR technology have primarily focused on fashion stores or consumer experiences. This study presented the characteristics of new virtual spaces created by media technology, and empirically determined the differences in fashion experiences in each virtual space. It followed a more detailed approach to the virtual experience and subdivided it. This study provides a basic framework for research on fashion content production and interactions with users in the Metaverse, which has been used increasingly in recent years. Therefore, this study makes an academic contribution by expanding the field of digital fashion design, which is still in its early stages, by narrowing the research gap.

The VR fashion show spaces of pataphysics provide creative motivation and aesthetic experiences of exploratory play. Unlike previous studies that focused on the environment of fashion stores (whose primary purpose is sales), and consumption behavior and intention, this study focused on fashion show environments. Current fashion shows are significant as they are communication channels that convey brand concepts and designers’ creativity. Experience is an important mechanism for improving professionalism (Dreyfus and Dreyfus, 1986), and the VR space can create a variety of experiences. Therefore, the current findings suggest that IVR fashion shows, and the spatial experience of pataphysics, could have an educational effect and help enhance the professionalism of students majoring in fashion, who are required to have abundant creative sensibilities. Future research should focus on its educational effects.

IVR technology changes the way that traditional fashion shows communicate. This study demonstrated that VR fashion shows play a positive role in fashion media by interacting with users, building a sense of closeness, and creating new fashion images. This is unlike previous fashion shows, which used a few unilateral methods of delivery. Unlike in the real world, in which new designers can be placed at a disadvantage when compared to luxury fashion brands that have a long history and tradition, VR fashion show spaces can give them the opportunity to effectively expand into new markets. Although physical fashion shows only last for about 20 min, there are concerns about the negative environmental effects they have, such as the vast amount of energy that is used and their heavy carbon footprint (Webb, 2022). Holding VR fashion shows can minimize their impact on the physical environment, and contribute to the development of a sustainable fashion industry by enhancing diversity in the industry, which is centered on mainstream fashion companies.

This study has some limitations. First, it did not adequately examine how technical problems in the devices used to experience VR fashion shows can affect a user’s fashion experience. Pallavicini et al. (2020) found that the images, sounds, and interactions of VR experience devices induced positive or negative reactions. Marsh et al. (2001) explained that the effect of sense of presence decreased and emotions such as discomfort and fear could be induced if interactions with the user were not smooth and the images on the device or screen were difficult to discern owing to issues such as slow buffering speeds. Some experienced problems with image quality owing to the Wi-Fi connection during the experiment, whereas some complained of discomfort owing to the weight of the device. Therefore, future research should exclude the technical limitations of the experience devices. Second, there could be differences in perception and cognition by sex (Rosa et al., 2014). Future research can evaluate differences based on sex and individual perceptions. Third, the experiment was conducted with fashion students; thus, there could be individual differences in students’ personalities and the results may not be generalizable to students in other fields. According to Dewey (2007), experience is formed by the interaction between the reciprocity of external conditions and subjective internal conditions. Thus, different dimensions of VR experience could be derived if other populations were targeted (e.g., consumers, other fashion professionals, or tourists).

## Data availability statement

The original contributions presented in the study are included in the article/supplementary material, further inquiries can be directed to the corresponding author.

## Ethics statement

The studies involving humans were approved by the Institutional Review Board in Changwon National University (no. 7001066-202301-HR-003). The studies were conducted in accordance with the local legislation and institutional requirements. The participants provided their written informed consent to participate in this study.

## Author contributions

SK: Conceptualization, Data curation, Formal analysis, Funding acquisition, Investigation, Methodology, Project administration, Resources, Supervision, Validation, Visualization, Writing – original draft, Writing – review & editing.
